# Adulteration of Medicines

**Published:** 1847-10

**Authors:** 


*Adulteration of Medicines.*—The following communications from the New-York College of Pharmacy, which we find in the New-York Journal of Medicine, together with the remarks by the Editor of that Journal, are deserving of serious attention. That frauds are practiced in the preparation of Medicines to an enormous extent, is not new to us. We have already commented upon the subject. The immediate consequences, as respect the treatment of disease, are of vast moment; and the remote evils in vitiating medical experience are of no inconsiderable importance. It is needless to amplify upon this view of the subject, for every medical man must at once perceive and appreciate its importance. We cannot see any other method by which the public and science are to be protected against these frauds, except by providing, under legal penalties, for the inspection of drugs by competent analysts. We have in previous remarks suggested this method. Our limits will not permit us to pursue the topic at this time.

"*Caution to Druggists.*—The Committee of Inspection of the College of Pharmacy, are instructed by the Board of Trustees to call the attention of Druggists to another dangerous fraud. A quantity of a base composition, under the name of Blue Pill is now in market, having been lately imported by, or consigned to and sold by Messrs. Cumming, Dodge and Co., of this city. It contains but little more than one-fifth the proper proportion of mercury, according to the examination of Prof. Reid, of this College, made at our request, that we might have the corroborating testimony of the best analyst in the city. His certificate of its composition, which we append, shows an extent of methodical depravity in the manufacture, against which honest dealers will have to oppose extreme vigilance in the inspection of what they buy.

The article under notice is put up in rather large, white, flat-covered jars, containing one pound each; the joint covered with coarse pink-colored muslin; white label with nothing upon it but the British arms and the words "Blue Pill," in rather heavy letters in blue ink. The mass has tin foil laid over it, under the earthen cover.

From what we learn of its history, this spurious compound was made by William Bailey, of Wolverhampton, whose manufacture of similar Blue Pill, two years ago, was so faithfully exposed by the late Mr. Adamson. A transcript of the correspondence on that occasion may be found in the American Journal of Pharmacy, Vol. XI., (New Series.) p. 148. Mr. Adamson's letter still remains unanswered.

GEO. D. COGGERSHALL,

JNO. H. CURRIE,

WM. HEGEMAN,

Committee of Inspection.

New-York, August 9th, 1847.

NEW-YORK HOSPITAL, Aug. 6th, 1847.

*Dear Sir.*—According to the request of the Inspection Committee of the College of Pharmacy, I have made an extended investigation into the composition of the Blue Pill furnished me, and have to report the following concerning this dangerous and heartless fraud.

Its composition by analysis is:

| | |
|---|---|
| Mercury, | 7·5 |
| Earthy Clay, | 27·0 |
| Prussian Blue, used in coloring, | 1·5 |
| Sand in combination with the clay, | 2·0 |
| Soluble saccharine matters, | 34·0 |
| Insoluble organic matters, | 12·0 |
| Water, | 16·0 |
| | 100 |

I could not see any thing differing in the state of combination of the mercury, from that generally found in Blue Pill.

The density of the Pill is about the same as the genuine. This is accounted for by the large quantity of earthy matter, which, in combination with the water, gives the requisite specific gravity, and makes the deception more plausible.

The presence of so much earthy matter furnishes us with an easy means of trying it. Place 100 grains on a clean iron plate or shovel, and place the shovel over the fire until the Pill is reduced to an ash. The genuine gives 2 per cent., or near it; this 29 per cent.

The per centage of mercury can be ascertained by a process proposed by me, and described in the American Journal of Pharmacy for 1844. Your's respectfully,

(Signed) LAWRENCE REID.

Mr. Geo. D. Coggeshall, Chairman of the Committee of Inspection of the College of Pharmacy.

*Remarks.*—This is but a sample of the numerous impositions practised upon American physicians in the manufacture and sale of drugs. We have again and again adverted to the frauds constantly carried on in the manufacture of spurious medicines, and have invited druggists and others conversant with these impositions to expose them through the medium of our pages. We have received in reply two or three letters, which have been published in former numbers of our Journal. We solicit still further communications on this subject.

It may not be generally understood, that the importation of drugs and medicines into this country, is chiefly in the hands of commission merchants, mostly foreigners, (German and French,) who are not druggists by profession, and who know nothing of medicines, except to buy cheaply and sell dearly. These men supply our wholesale dealers, who, for the most part, have nothing to do with the importation of the articles in which they deal, and who are not unfrequently imposed upon, as in the case of Blue Pill, as above stated. The commission dealers have agents, travelling and resident, abroad, who buy up the refuse drugs in all the principal European cities, and send them to this country, where they generally meet with a ready sale. We may mention, for example, *Rhubarb*, of which, we are credibly informed, there have been but two invoices of a good article (*Turkey*) brought into this market since December. Immense quantities, however, have been imported, of a worthless, worm-eaten article, called *Turkey*, invoiced from *two pence to eight pence sterling*, from *four to sixteen cents per pound*, which, we have reason to know, has been ground and sold to our retail druggusts for genuine Turkey Rhubarb, worth four or five shillings a pound. The *Compound Extract of Colocynth*, which has been imported into this market for the last year, does not contain a particle of Colocynth, but is made up of an inferior sort of Aloes, with some other worthless ingredients. A great proportion of the Compound Extracts are adulterated in like manner. More than half of the narcotic and other extracts, as of *Belladonna*, *Conium*, *Hyoscyamus*, *Aconite*, *Rhatany*, etc., are entirely destitute of any active properties, as we know from our experience, and *Opium* is now rarely to be met with in a genuine form. The *Attar of Roses* is more frequently than otherwise adulterated with the oil of Rhodium, of which there is also an artificial compound prepared for this very purpose. Our *Volatile Oils* are adulterated more than half with sweet and other cheap oils. The *Hydrargyrum Ammoniatum*, U. S. P. *White Precipitate*, of *Bailey's* manufacture, (Wolverhampton,) is now as much adulterated as the sample of *Blue Pill* from the same house, analyzed by Prof. Reid. This is an article of a chemical nature, which should, if prepared according to the Pharmacopœia, always be of the same quality; and yet we have its invoice price ranging from three to six shillings sterling per pound, *according to quality*, We have not ascertained whether it is mixed with *clay*, like the blue pill, *white lead*, *chalk*, or *gypsum*, but we have no doubt that one of these will be found to constitute more than 50 per cent. of it. whenever an analysis may be made. An article is now imported, under the name of *Colocynth Powder*, which is probably *Colocynthin*, mixed with some inert vegetable powder; this varies in our Custom-House invoices, from 5 to 14 shillings sterling per lb., and is often two-thirds adulterated. The *Extract of Rhubarb*, from 4 to 9 shillings sterling per pound, according to quality. The *Extract of Sarsaparilla*, as now imported, is a worthless imposition. *Quinine* is now imported in bulk instead of bottles. These latter are now generally manufactured here, together with the *labels*, according to the latest French patterns, usually the *Pelletier* stamp, we believe is preferred. The *Quinine* now generally in use all over this country, is at least one-half *Salacine;* this latter being imported very extensively for this purpose, at an expence of less than one-third that of quinine. Some dealers, however, use flour or starch for the same purpose. We believe that it is owing to the adulteration of this article that such *large doses* are required, and *safely borne*, in the malarious diseases of the South and West. We have known practitioners in these regions occasionally get hold of a genuine article, and they very soon found that their patients, so far from requiring a *drachm*, or even half that quantity, found from five to fifteen grains sufficient. The house of *Teschdorf, Fischer & Co.*, of Hamburgh, send us immense quantities of drugs of every description, especially of *Extracts*, as of "*Carduuss Benedictus*," "*Chelidonium*," "*Fumaria*," "*Gratiolus*," "*Lactuca Virosa*," *Millefolia*," and "GRAMINIS"! Where are these articles used? What are the medicinal properties of the *Extract of Grass*? The only use for the latter, we have very good reason to believe, is to mix with genuine extracts, for the purpose of dilution. The invoice price of these extracts varies from forty cents to $1,75 per pound.

Much of the *Nitrate of Silver*, so called, now on sale in our wholesale drug establishments, does not contain a particle of the metal; whether the substitution is prepared here or abroad, we do not know. Of the *Hydriodate of Potash* also a large proportion is utterly worthless, *Iodine* not entering into its composition; the article is extensively imported in this shape. In order to have an article on which they can depend, we would recommend physicians everywhere, to prepare their own Hyd. of Potash' which can be readily done as follows:—Heat slightly a mixture of 100 grains of Iodine, 2 drachms of water, 75 grains of carbonate of potash, with 30 grains of iron filings. The mass is dried to redness. The resulting red powder is heated with water, then filter and evaporate to dryness; 109 parts of Iodine will thus furnish 135 parts of every white iodide of potassium, but slightly alkaline.

Thus we could go through with the whole catalogue of medicines in daily use by the physicians. It is now well known that there are establishments abroad for the express purpose of manufacturing spurious drugs for the American market, and it is high time that something was done to put a stop to it. As one important step towards reform, we hope that our wholesale dealers will hereafter import their own medicines, and not trust to a set of sharpers, who think more of money than they do of life and health. There is no propriety in leaving this branch of business in the hands of men who are not competent judges of the genuineness of the articles in which the deal. In the next place, we hope Congress will, at their next session, pass a law, forfeiting all spurious and adulterated drugs, and subjecting the owner or consignee to heavy penalties. We have appraisers now connected with the Custom-House, who are regularly educated physicians and chemists, and who are fully competent to detect these impositions, whenever they may be praetised. At present, although the government is fully aware of these extensive frauds, it has no power whatever to prevent them; its *ad valorem* estimation may be *nothing*, or next to nothing, as in the case of the rhubarb, apprised *in the invoice* at two pence sterling per pound; but it has no right to exclude the article from aur markets. We need a stringent law, to prevent such practices in future. Again, physicians must purchase their medicines in the crude state, and not in powder; if they do, they will be imposed upon, nine times out of ten. They must take their own extracts, syrups, pills, and tinctures. They must resort more frequently to the use of our indigenous medicines, and never employ a foreign article where a domestic one will answer the purpose. When they do purchase, they should buy only of those wholesale dealers who import their own stock; and not take their articles from those who are unacquainted with the characters of genuine drugs. And lastly, they should deal only with those who sustain the reputation of being *honest men*, and whose consciences would not allow them to go on quietly in the daily practice of imposture and deception, involving the lives and health of their fellow-men. We hope the "New-York College of Pharmacy" will pursue this subject, and expose a few more of the frauds now practised in the manufacture and sale of medicines. And although we are not personally acquainted with the Hon. Secretary of the Treasury, R. J. Walker, Esq., we have reason to believe that he will cheerfully co-operate in bringing about a reform in this matter, and thus put a check to the importation of spurious and adulterated articles, which not only detract largely from the public revenue, but prove destructive to the lives and health of our citizens, and often fatal to the reputation of the regular practitioner of medicine.

[Since the above was written, and in the hands of the Printer, we have received the following communication from the New-York College of Pharmacy.—Ed.]

New-York, August 24th, 1847.

Sir.—In behalf of the College of Pharmacy of the city of New-York, I have the honor to submit for your consideration, and insertion in your valuable Journal, the proceedings of the Board of Trustees, in relation to the importation from Europe of large quantities of sophisticated pharmaceutical and chemical preparations, which must often prove highly injurious to those who may be subjected to their use.

The College has, for many years, exerted all its influence to oppose this system of culpable speculation, by cautioning dealers, through the public prints, against the purchase of such articles as were proved by careful analysis to be dangerous. This it has done cheerfully and fearlessly.

These efforts having proved insufficient wholly to suppress this alarming evil, the College has resolved to ask the co-operation of the other Colleges of Pharmacy, and all the medical institutions and practitioners in both branches throughout the Union, in an application to Congress for a law, declaring that all pharmaceutical preparations and chemicals which shall be found, upon careful examination, to be spurious, shall be confiscated and destroyed.

With the assurance of my perfect esteem,

I remain your obedient servant,

JOHN MILHAU, Pres. Coll. of Phar. of N. Y.

To Chas. A. Lee, M. D., Editor N. Y. Journal of Medicine.

At a special meeting of the Board of Trustees of the College of Pharmacy of the city of New-York, held on August 9th, 1847, convened for the express purpose of taking into consideration the best measures to prevent the introduction, throughout the United States, of sophisticated ans misnamed Chemical and Pharmaceutical preparations—it was unanimously

*Resolved*, That the officers of this institution be requested forthwith to call the attention of the Secretary of the Treasury of the United States to the fact, that large quantities of spurious medicinal preparations are being introduced daily into this country, not only to the prejudice of the Custom-House revenue and the honest importer, but in the sequel jeopardizing the health and lives of all those who require medical aid, throughout the land. That the Secretary of the Treasury be respectfully requested to apply the most stringent regulations within his power, to check this alarmingly growing evil.

It was further *Resolved*, That the Philadelphia College of Pharmacy, and other Colleges of Pharmacy and Medicine, be officially requested to unite with us in presenting a memorial to Congress, to devise means to suppress this most dangerous fraud, by making all such sophisticated articles liable to forfeiture.

JOHN MILHAU, President.

OLIVER HULL, GEO. D. CONGESHALL, WM. L. RUSHTON, } Vice Presidents.

JOHN SNOWDEN, Sec.

J. S. ASPINWALL, Tr.

